# Draft Genome Sequence of *Staphylococcus sciuri* subsp. *sciuri* Strain Z8, Isolated from Human Skin

**DOI:** 10.1128/genomeA.00714-15

**Published:** 2015-07-23

**Authors:** Xinjun Hu, Beiwen Zheng, Haiyin Jiang, Yi Kang, Qing Cao, Huibin Ning, Jia Shang

**Affiliations:** a Department of Infectious Diseases, Henan Provincial People’s Hospital, Zhengzhou, China; b State Key Laboratory for Diagnosis and Treatment of Infectious Disease, the First Affiliated Hospital, Zhejiang University, Hangzhou, China

## Abstract

*Staphylococcus sciuri* subsp. *sciuri* strain Z8 was isolated from a skin wound infection of a patient with infective endocarditis. To the best of our knowledge, the genome sequence of the species *S. sciuri* has not been previously studied. The complete genome sequence of strain Z8 includes a genome of 2,620,868 bp (32.43% GC content) without any plasmids.

## GENOME ANNOUNCEMENT

*Staphylococcus sciuri* was first described by Kloos et al. in 1976 (1). Members of this group are widespread in nature. Many studies have reported the frequent isolation of *S. sciuri* from various sources, including foods, rodents, and marsupials and occasionally from humans (2). This organism may be responsible for endophthalmitis (3), peritonitis (4), endocarditis (5), urinary tract infections (6), pelvic inflammatory disease (7), septic shock (8), and wound infections (9). Strain Z8 was isolated from a skin wound infection of a patient with infective endocarditis. It is a multidrug-resistant bacterium, resistant to benzylpenicillin, oxacillin, erythromycin, tetracycline, trimethoprim, and clindamycin. We determined the genomic sequence of the strain because of the clinical relevance of this group*.*

Strain Z8 was grown aerobically on Columbia blood agar base, at 37°C for 24 h. Genomic DNA was extracted using the DNeasy blood and tissue kit (Qiagen, Germany). The quantity of DNA was measured by the Cubit. Then, 10 µg of DNA was sent to Zhejiang University.

One DNA library was generated (422-bp insert size, with the Illumina adapter at both ends), and then sequencing was performed by using an Illumina HiSeq 2000 genomic sequencer with a 2 × 100 paired-end sequencing strategy. A total of 1,531 Mbp clean-filtered reads were assembled into scaffolds using Velvet version 1.2.07 (10); then, we used a PAGIT flow (11) to prolong the initial contigs and correct sequencing errors. Predicted genes were identified using Glimmer version 3.0 (12); tRNAscan-SE version 1.21 (13) was used to find tRNA genes, whereas ribosomal RNAs were found by using RNAmmer version 1.2 (14). To annotate predicted genes, we used HMMER version 3.0 (15), and the KAAS server (16) was used to assign translated amino acids into KEGG orthology with SBH (single-directional best hit) method. Translated genes were aligned with the COG database using NCBI BLASTp.

The draft genome sequence of strain Z8 revealed a genome size of 2,620,868 bp and a G+C content of 32.43% (107 scaffolds with an *N*_50_ of 66,138 bp). These scaffolds contain 2,614 coding sequences (CDSs), 42 tRNAs (excluding 1 pseudo-tRNA), and incomplete rRNA operons (0 small subunit rRNA and 2 large subunit rRNAs). A total of 600 protein-coding genes were assigned as putative function or hypothetical proteins, and 2,161 genes were categorized into COG functional groups (including putative or hypothetical genes).

*S. sciuri* is increasingly reported as a cause of infection in humans. To the best of our knowledge, the genome of *S. sciuri* has not been reported. The availability of the *S. sciuri* subsp. *sciuri* strain Z8 genome could prompt the development of molecular tools to characterize its pathogenesis and information for drug resistance mutations.

### Nucleotide sequence accession number.

This whole-genome shotgun project has been deposited at DDBJ/EMBL/GenBank under the accession number JANE00000000. The version described in this paper is the first version.
